# Exploring Cu-Doping for Performance Improvement in Sb_2_Se_3_ Photovoltaic Solar Cells

**DOI:** 10.3390/ijms232415529

**Published:** 2022-12-08

**Authors:** Giulia Spaggiari, Danilo Bersani, Davide Calestani, Edmondo Gilioli, Enos Gombia, Francesco Mezzadri, Michele Casappa, Francesco Pattini, Giovanna Trevisi, Stefano Rampino

**Affiliations:** 1 Institute of Materials for Electronics and Magnetism (IMEM), Consiglio Nazionale delle Ricerche, Parco Area delle Scienze 37/A, 43124 Parma, Italy; 2 Department of Mathematical, Physical and Computer Sciences, University of Parma, Parco Area delle Scienze 7/A, 43124 Parma, Italy; 3 Department of Chemistry, Life Sciences and Environmental Sustainability, University of Parma, Parco Area delle Scienze 11/A, 43124 Parma, Italy

**Keywords:** Sb_2_Se_3_, thin-film solar cells, Cu doping, pulsed electron deposition

## Abstract

Copper-doped antimony selenide (Cu-doped Sb_2_Se_3_) thin films were deposited as absorber layers in photovoltaic solar cells using the low-temperature pulsed electron deposition (LT-PED) technique, starting from Sb_2_Se_3_ targets where part of the Sb was replaced with Cu. From a crystalline point of view, the best results were achieved for thin films with about Sb_1.75_Cu_0.25_Se_3_ composition. In order to compare the results with those previously obtained on undoped thin films, Cu-doped Sb_2_Se_3_ films were deposited both on Mo- and Fluorine-doped Tin Oxide (FTO) substrates, which have different influences on the film crystallization and grain orientation. From the current-voltage analysis it was determined that the introduction of Cu in the Sb_2_Se_3_ absorber enhanced the open circuit voltage (V_OC_) up to remarkable values higher than 500 mV, while the free carrier density became two orders of magnitude higher than in pure Sb_2_Se_3_-based solar cells.

## 1. Introduction

The search for reliable alternatives to the currently leading absorber materials for thin-film photovoltaic solar cells is very active. In order to replace silicon (a-Si), cadmium telluride (CdTe), and copper indium gallium diselenide (CIGS), researchers are exploring materials that are cheaper, contain low-toxic and earth-abundant elements and have a high conversion efficiency [1,2,3,4].

In this context, the interest in Antimony Selenide (Sb_2_Se_3_, or “ASe”) has constantly grown in the last few years [5]. Sb_2_Se_3_ comprises the following properties: (i) 1.0–1.2 eV energy gap, leading to a theoretical photon conversion efficiency (PCE) of 32.2%, according to the Shockely–Queisser model; (ii) very high density of states near the valence band maximum; (iii) a very high absorption coefficient (that efficiently compensates a just “decent” carrier mobility), and (iv) a peculiar crystal structure that allows its film grains to be almost dangling, bonds-free [6,7].

The ASe crystalline structure is essentially made of 1D ribbons of (Sb_4_Se_6_) units stacked along the c-axis of an orthorhombic cell (*Pbnm* space group), with covalent bonds within the 1D chain and only weak van der Waals bonds between these parallel ribbons, indicating that most of its physical properties are strongly anisotropic. The main reason why Sb_2_Se_3_ has been known since 1950 but only recently been considered for photovoltaic application is that it often grows in thin films, with 1D ribbons parallel to the substrate, resulting in a very poor and hopping-dominated vertical transport across the film. Starting from 2014, new deposition processes were successfully tested [6,7,8,9,10,11,12,13,14] and, pushed to the results first achieved by the closed space sublimation (CSS) technology, producing Sb_2_Se_3_ films with properly oriented ribbons [15,16], the efficiency of Sb_2_Se_3_-based solar cells rapidly increased from <1% up to the current 10.57% record [9]. Despite this significant improvement, there are still two main issues to be solved to enhance the efficiency of ASe-based solar cells [5,17]. On the one hand there is still a wide margin for improvement in the choice of materials for each layer composing the cell, not only for the optimization of bands alignment [17], but also for the minimization of the interface defect density and of the surface recombination rate, that are often reported to be very high for Sb_2_Se_3_ and traditional buffers such as CdS [5,6]. On the other hand, even on the most efficient ASe-based cells, the reported V_OC_ (open circuit voltage) and FF (fill factor) values are generally too low and likely limited not only by the presence of deep defects that leads to the short carrier lifetime, but also by the small built-in potential due to the very low hole density (≈10^13^ cm^−3^) in undoped Sb_2_Se_3_ [5,6,7].

In addition to the possibility of improving the Sb/Se ratio, especially through selenization post-grow treatments [18,19], which can effectively reduce the density of intrinsic deep defects such as V_Se_ and Sb_Se_, several attempts to dope Sb_2_Se_3_ with different elements have been reported in the literature, both from a theoretical point of view and experimentally. More or less effective p-type conductivity enhancements have been reported or predicted by doping with Pb [20,21], Sn [22,23], Mg [24], N [25], and a low concentration of Bi [26]. However, since Bi and Sb belong to the same group, Bi substitution mainly affects the Sb/Se ratio and the concentration of intrinsic defects. Indeed, a higher concentration of Bi leads to n-type conductivity [26,27]. Also, Te, which is homo-valent to Se, was reported to act mainly by affecting the Se/Sb ratio [28]. On the contrary, Fe [24,29], I [22,30] or other halides [23] are reported to induce only n-type conductivity.

Cu doping was reported to be promising for the enhancement of p-type conductivity (e.g., see [23]), but to the best of our knowledge it has been obtained only with very high trap density (that adversely affected V_OC_, limiting it below 300 mV) [30] or by a post-growth treatment of the surface with CuCl_2_, which induced an n-type inversion at grain boundaries [31]. Otherwise, the use of Cu with Sb and Se was mainly intended to form the CuSbSe_2_ compound [32,33,34]. As far as it is reported in the literature, there are no scientific works yet reporting the deposition of Cu-doped ASe films by pulsed deposition techniques.

In this work we presented the main properties of Cu-doped Sb_2_Se_3_ thin films, starting from a Cu-doped ASe solid target ablated by low-temperature pulsed electron deposition (LT-PED). Structure, morphology, and composition of the obtained samples were characterized. Particular attention was paid to the orientation of 1D-ribbons on the substrate as a function of the deposition parameters and the substrate (Mo and FTO, fluorine tin oxide). The electrical characterization of the Cu-doped ASe films and the photovoltaic cells were compared to those obtained on undoped Sb_2_Se_3_ films deposited both by PED [14] and magnetron sputtering (MS) [10], whose performances were mainly limited by low V_OC_ values below 300 mV. This study confirmed the role of Cu as a dopant able to effectively enhance free carrier density.

## 2. Results

### 2.1. Tuning of LT-PED Deposition Parameters

Cu, whose main oxidation states are +1 and +2, was chosen for enhancing the p-type conductivity of Sb_2_Se_3_ (Sb oxidation state is +3). Therefore, Cu_2_Se and CuSe are the stable compounds for Cu and Se. Moreover, as mentioned before, a CuSbSe_2_ phase also exists. Since the crystal structures of all these compounds are different from the one of Sb_2_Se_3_, it is predictable that the Sb substitution with Cu has a threshold above which different phases can segregate.

**Figure 1:**
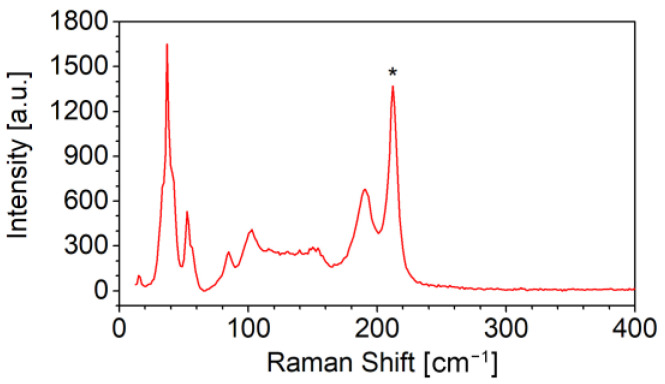
Raman spectrum collected on a local spot of a thin-film deposited by LT-PED with a Sb_1.5_Cu_0.5_Se_3_ target, where CuSbSe_2_ phase was also present, as revealed by the peak at ~210 cm^−1^ (highlighted with * symbol). All the other main peaks in the spectrum are typical of a Sb_2_Se_3_-like phase [36].

Hence, polycrystalline targets with different Cu contents were prepared and tested for the deposition of Cu-doped ASe film by LT-PED, and micro-Raman spectroscopy was performed on different points on the films obtained with these targets. On films with a composition with up to 5% of Cu (atomic percentage on the whole stoichiometry, corresponding to Sb_1.75_Cu_0.25_Se_3_) each local Raman measurement spectrum still displayed only the peaks associated to the Sb_2_Se_3_ phase. On films with 10% of Cu (atomic percentage on the whole stoichiometry, i.e., Sb_1.5_Cu_0.5_Se_3_) some peaks from CuSbSe_2_ phase were occasionally detected. One of these spectra is reported in Figure 1. While the Sb_2_Se_3_ spectrum is generally characterized by an intense peak at about 190 cm^−1^ from A_g_ modes and a weaker shoulder in the 210–215 cm^−1^ range from B_1g_ modes [35], the intense peak at about 212 cm^−1^, indicates the presence of CuSbSe_2_ [36]. Therefore, the target with 5% of Cu was chosen for all the following experiments: this is the same concentration reported in other works in which Cu doping was obtained from the liquid phase [24].

**Figure 2:**
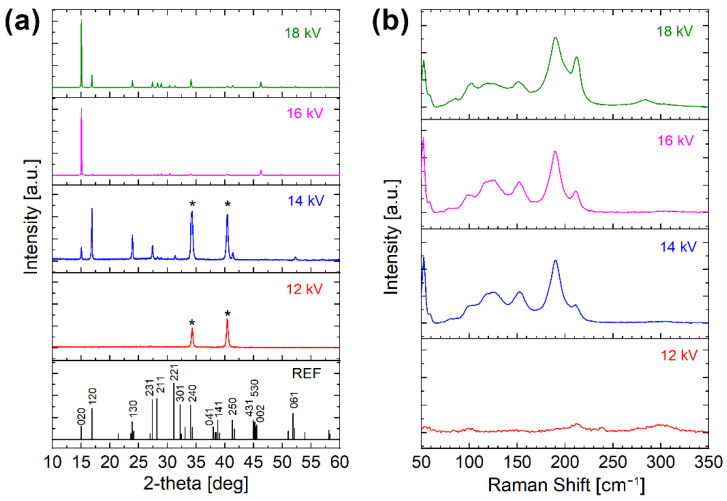
Structural characterization of Cu-doped Sb_2_Se_3_ thin films deposited by LT-PED on Mo substrate with different accelerating voltages: (**a**) XRD patterns, with indexed reference (JCPDS 15–0681) for undoped Sb_2_Se_3_ at the bottom (* indicates Mo substrate); and (**b**) Raman spectra.

A preliminary study was performed by using different values for the LT-PED accelerating voltage, which is known to strongly affect several aspects of growth, such as the film composition and crystallization [14]. Cu-doped ASe thin films were deposited on an Mo substrate at 12 kV, 14 kV, 16 kV and 18 kV, keeping constant all the other parameters but the deposition time, in order to obtain films with comparable thickness. The structural characterization by X-ray diffraction (XRD) and Raman spectroscopy of the obtained film samples is reported in Figure 2.

The film deposited at 12 kV was amorphous and only XRD peaks from the substrate are visible in the plot, while the film deposited at 14 kV was better crystallized and diffraction peaks similar to those of undoped Sb_2_Se_3_ can be easily distinguished. Unfortunately, the relative peaks intensities reveal that the growth direction of the 1D ribbons is mainly parallel to the substrate since peaks with *hk*0 indexes (*l* = 0) are predominant. The films grown at 16 kV and 18 kV exhibit even stronger *hk*0 preferential orientations, with a very high peak for the 020 reflection, in full agreement with the undoped ASe thin films deposited by LT-PED on a Mo substrate [14]. A few peaks cannot be assigned to a Sb_2_Se_3_-like structure (e.g., those at 29.0° and 46.3°), probably related to some Cu-rich phases, are also present at high acceleration voltage.

Figure 2b confirms and completes these results, since the Raman spectrum of the film deposited at 12 kV is also typical of an almost amorphous film with weak and very wide peaks similar to those of CuSbSe_2_, while the spectrum of the film deposited at 14 kV is analogous to the one of undoped Sb_2_Se_3_. Similar Raman spectra are obtained for the films deposited at 16 kV and 18 kV, indicating that the dominant structure is a Sb_2_Se_3_-like, and the peak at about 210 cm^−1^ can be assigned to the Sb–Se vibration mode.

**Figure 3:**
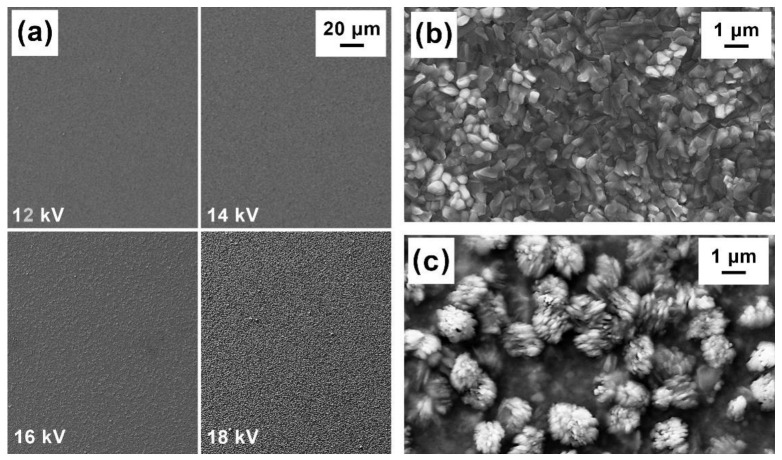
SEM images of Cu-doped Sb_2_Se_3_ thin films deposited by LT-PED on Mo substrate: (**a**) a surface roughness comparison for samples deposited with different accelerating voltages; (**b**) a higher magnification of the sample deposited at 16 kV; and (**c**) a higher magnification of the sample surface deposited at 18 kV.

The same samples were also characterized by scanning electron microscopy (SEM) and energy dispersive spectroscopy (EDS), for the study of their morphology and composition. The SEM images of the films’ surface shown in Figure 3 display an increase in grain size with the LT-PED accelerating voltages, with bunches of needle-like crystals emerging from the surface for the sample grown at 18 kV (Figure 3c).

EDS microanalysis revealed that the Cu amount in the thin films deposited at 12 kV and 14 kV is only 1–2%, rising to about 5% (as in the target) in those deposited at 16 kV and 18 kV. The effect of the acceleration voltage on the film stoichiometry can be explained by referring to other works on pulsed electron deposition of chalcopyrites [37,38]. At low acceleration voltage, the electron beam mainly impinges on the first layers of the target surface, thus promoting the thermal evaporation of the most volatile species from the latter. For this reason, Cu, which is the element with the highest melting-point, was not stoichiometrically transferred. At higher acceleration voltages, the pulsed electron beam is able to penetrate under the target surface, promoting the ablation of the target species and the stoichiometric transfer of these latter on the substrate.

These studies have been repeated even on ASe films grown on FTO substrate. The effects of the acceleration voltage on the film stoichiometry are not affected by the substrate: both on Mo and FTO, the deposition at 16 kV seems to offer the better compromise, because despite the not-ideal *hk*0 preferential orientations, it produced thin films with the highest Cu concentration and no evidence of other phases. This value of the acceleration voltage was hence chosen for the following experiments.

### 2.2. Role of the Substrate

It has been widely reported in the literature, as well as by this group, that the substrate plays a key role in the orientation of the 1D ribbons in the film grains [6,13,14]. The most common but also undesired orientations are those that increase the intensity of *hk*0 peaks in the corresponding XRD patterns since they are related to grains with preferential growth orientation with the c-axis in the same plane of the substrates.

A more immediate representation of the average orientation can be offered by a “texture coefficient” (TC), defined as:

TC(*hkl*) = {[*I*(*hkl*)/*I*_0_(*hkl*)]/Σ*_n_*[*I*(*h’k’l’*)/*I*_0_(*h’k’l’*)]} ∙ 100% (1)

where *I*(*hkl*) is the relative intensity of the (*hkl*) peak in the collected XRD pattern and *I*_0_(_hkl_) is the relative intensity for the same peak in the JCPDS 15-0681 reference. In the denominator, the summation is calculated with the same ratio for *n* = 10 selected reflections, chosen among the strongest and most frequently observed ones: (020), (120), (130), (231), (211), (221), (041), (141), (002) and (061). Since *n* here is 10, a preferential orientation along the *hkl* direction is present when TC(*hkl*) > 10%.

For undoped Sb_2_Se_3_ films deposited by LT-PED, it was previously reported [14] that Mo substrate produced the worst orientation for photovoltaic application, having mostly grains with a preferential *hk*0 orientation. Also, in the case of Cu-doped Sb_2_Se_3_ films deposited on Mo at 16 kV, the sum of TC values for *hk*0 peaks reached 98.8%.

**Figure 4:**
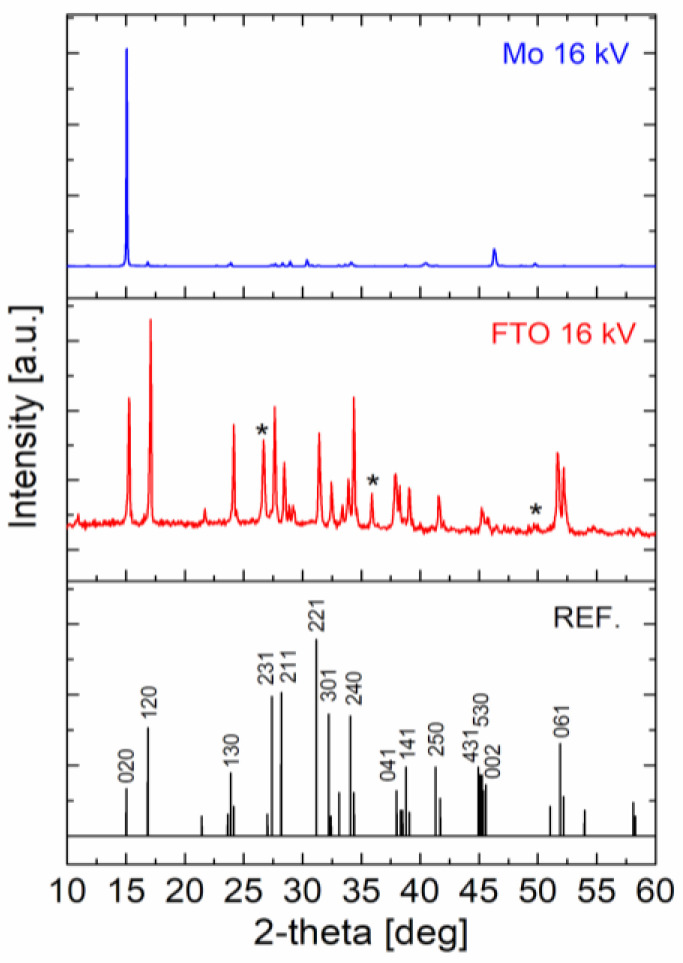
XRD patterns of Cu-doped Sb_2_Se_3_ thin films deposited by LT-PED at 16 kV on Mo and FTO substrates; indexed reference (JCPDS 15-0681) for undoped Sb_2_Se_3_ is reported at the bottom; * indicates the FTO substrate.

For comparison, Cu-doped Sb_2_Se_3_ thin films were deposited at 16 kV also on an FTO substrate, which shows a better influence on the ribbon orientation. Indeed, the XRD clearly indicates that in this case there is not a preferential orientation for in-plane directions; the corresponding sum of TC values for *hk*0 peaks is in this case as low as 58.7%, as reported in Figure 4.

### 2.3. Electrical Characterization of Thin films and Cells

Photovoltaic cells were then made with the Cu-doped ASe thin films, following the same procedure described in Ref. [14]. The complete structure for the two kinds of cells was the following: AZO/ZnO/CdS/Cu:Sb_2_Se_3_/Mo/Glass and AZO/ZnO/CdS/Cu:Sb_2_Se_3_/ FTO/Glass. The optimization of the cells’ layers was beyond the scope of this work, so, despite these layer structures probably not being the ideal choice for a top-efficiency cell, they were chosen for comparing the results with those previously reported for the undoped absorber [14].

The electrical properties of Cu-doped ASe solar cells were investigated. Capacitance–Voltage (C-V) measurements were carried out to determine the net acceptor concentration (N_A_), according to the following equation:

1/C^2^ = 2(V_b_ − V)/qA^2^εN_A_ (2)

where ε is the Sb_2_Se_3_ relative permittivity, A is the cell area, V_b_ is the built-in voltage of the junction.

**Figure 5:**
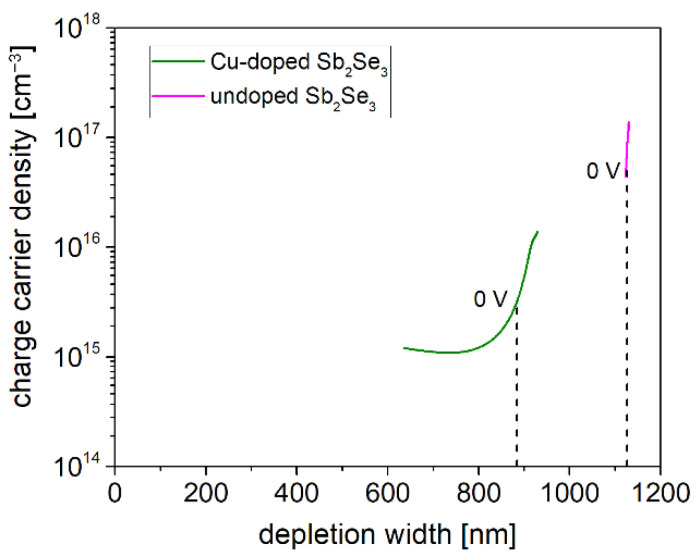
Free carrier profiles obtained from the d(1/C^2^)/dV at 120 K on undoped and 5% Cu-doped Sb_2_Se_3_ solar cells. The test signal frequency and amplitude are 1 MHz and 35 mV, respectively.

Figure 5 shows the free carrier profiles obtained from the d(1/C^2^)/dV at 120 K of two solar cells with undoped and 5% Cu-doped ASe absorber layers, respectively. While the undoped ASe layer, with a thickness of 1100 nm, seems to be fully depleted, from the minimum of the typical u-shaped curve [39] a rough estimation of the Cu-doped Sb_2_Se_3_ free carrier concentration is estimated in the order 10^15^ cm^−3^, while the depletion layer at V = 0 V is <900 nm. Considering that Sb_2_Se_3_ is naturally an almost intrinsic semiconductor with a very low free carrier density (10^13^ cm^−3^) [6], this means that the free carrier concentration was successfully increased by about two orders of magnitude with respect to the undoped ASe films thanks to Cu doping.

**Figure 6:**
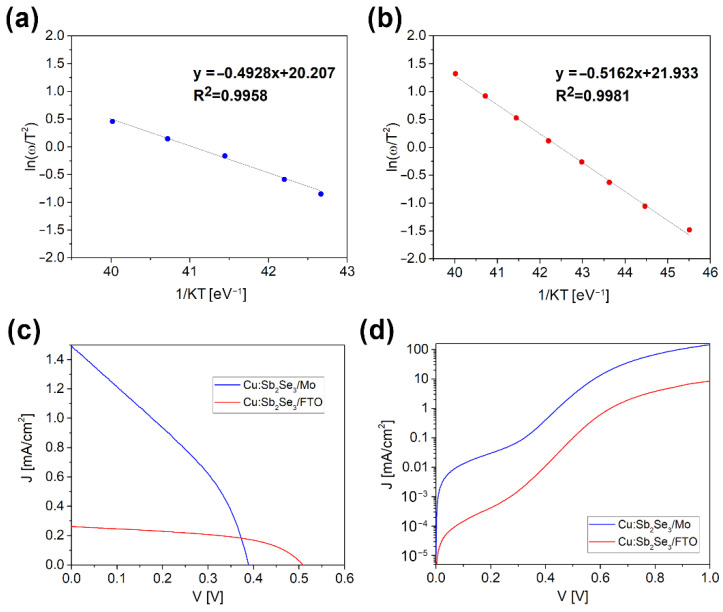
Electrical characterization of the two cells with Cu-doped Sb_2_Se_3_ on Mo (Glass/Mo/Cu:Sb_2_Se_3_/CdS/ZnO/AZO) and on FTO (Glass/FTO/Cu:Sb_2_Se_3_/CdS/ZnO/AZO) with the highest recorded Voc values: Arrhenius plots of ln (w/T^2^) vs 1/KT calculated from the C vs w spectra in the 250K-300K temperature range for the cell on Mo (**a**); and on FTO (**b**). J-V plot for the two cells under illumination (**c**); and in the dark (**d**).

From the thermal admittance spectroscopy (TAS) measurements, it was determined that the activation energy, E_A_, of the level responsible for the main capacitance step in the C vs **ω** plots, carried out at different temperatures, (**ω** = 2**π**ƒ, ƒ = frequency of the test signal) is E_A_ = 0.505 ± 0.015 eV in both cells (Figure 6a,b), independent of the back contact nature. This value is in good agreement with the results of TAS experiments on other Sb_2_Se_3_ solar cells, which attribute this mid-gap energy level to a ASe/CdS interface or near-interface defect [40,41].

The current–voltage (J-V) characteristics of the two cells made with Cu-doped ASe thin films on the different substrates with the highest recorded V_OC_ values are reported in Figure 6c,d (under illumination and dark measurement, respectively). By comparing the values with the undoped films, V_OC_ raised from 315 mV to 388 mV for the cell with Mo substrate and from 256 mV to the remarkable value of 509 mV in the case of FTO substrate. This value is among the three highest ever reported for a Sb_2_Se_3_-based solar cell [17,18,40]. The short-circuit current density, J_SC,_ also slightly increases for the cell on the Mo substrate, from a very low 0.3 mA/cm^2^ to 1.5 mA/cm^2^, while unfortunately it decreased for the cell on the FTO substrate from 25.6 mA/cm^2^ to 0.3 mA/cm^2^, undermining the increase in the FF from 39.2% to 51.9%. The ideality factors calculated at room temperature from the dark I-V characteristics according to the method proposed by Hegedus [42] are typically around 1.8–1.9 in both types of solar cells, indicating that the saturation current is dominated by SRH (Shockley–Read_Hall) recombination in the space charge region (Figure 6d). The electrical parameters of both type of cells are reported in Table 1.

**Figure 7:**
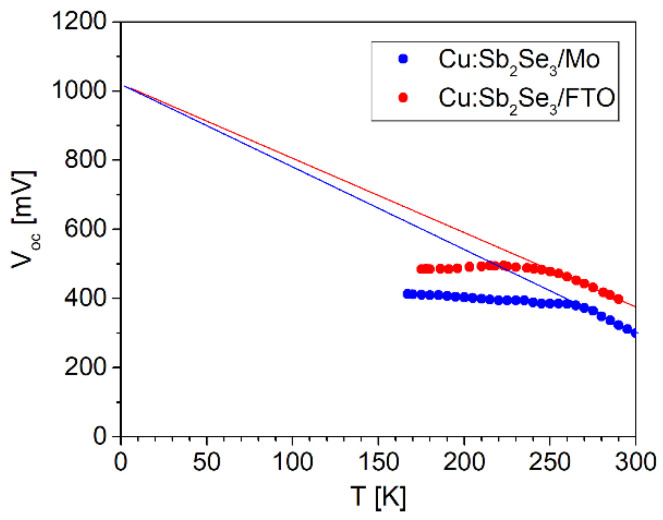
Temperature dependence of V_OC_ for Cu-doped Sb_2_Se_3_ tested cells. The extrapolated value of V_OC_ as T→0 is indicated by the dashed line (≈1.02 V).

In the case of FTO, as well as the larger shunt resistance, R_sh_, with respect to Mo, a series resistance, R_s_, close to 5 Ω cm^2^ has been observed, and its origin is probably related to a non-ohmic interface between n-type FTO and doped p-type Sb_2_Se_3_, which could also explain the huge current drop. From the open circuit voltage vs temperature plot of Cu-doped ASe based solar cells grown on MO/FTO substrates (Figure 7), it was determined that the T = 0 K intercept indicates an energy barrier for recombination of approximately 1.02 eV. As this value is near to the bandgap-energy of the Sb_2_Se_3_ absorber, the obtained result suggests that the recombination in the bulk of the absorber is the dominant mechanism.

## 3. Discussion

Sb_2_Se_3_ is an almost intrinsic semiconductor, with a very low free carrier density (10^13^ cm^−3^), insufficient to provide a good photovoltaic performance. Cu proved to be effective in increasing both the free carrier density, up to a value that is more suitable for a photovoltaic cell (10^15^ cm^−3^), and the value of V_OC_, up to over 500 mV, which is closer to the theoretical simulations for this material when the 1D ribbons in its structure are not mainly lying parallel to the substrate.

As expected from previous results on solar cells made with undoped Sb_2_Se_3_ deposited by LT-PED, the AZO/ZnO/CdS/Cu:Sb_2_Se_3_/FTO/Glass cell gave the best result, not only in terms of V_OC_ (509 mV), but also of ribbons orientation. On the contrary, the counterproductive preferential *hk*0 orientation of ribbons in the Cu-doped Sb_2_Se_3_ film deposited on Mo, hindered the improvement of V_OC_ value (388 mV) in the AZO/ZnO/CdS/Cu:Sb_2_Se_3_/FTO/Glass cell.

On the other hand, the very low J_SC_ values measured in the cell grown on FTO confirmed that the present layers architecture is not effective for Cu-doped Sb_2_Se_3_. The cell architecture, indeed, derived from the typical CIGS-based solar cells, is far from being optimized for this new alternative material. Since the film properties seem to be good enough, the origin of the low J_SC_ and not optimal Rs value (5 Ω cm^2^) is probably attributable to the formation of an undesired barrier at the interface between the p-type Cu-doped ASe film and the n^+^-type FTO back-contact. A comprehensive study of electrical properties highlights the presence of deep levels across the junction. More specifically, a deep level with an activation energy of about 0.51 eV is present in both cells with Mo and FTO substrates and it is probably related to defects at the CdS/Sb_2_Se_3_ interface.

While the optimization of the architecture of the photovoltaic device goes beyond the scope of this work, this work demonstrates the potential of Cu-doping for the improvement of Sb_2_Se_3_-based solar cells.

## 4. Materials and Methods

One-inch-wide Cu-doped Sb_2_Se_3_ targets were synthetized starting from the proper amount of 6N purity Cu, Sb and Se elements (Alfa Aesar, Kandel, Germany), by radiofrequency induction heating in a semi-closed quartz crucible. The results reported here are referred to targets with the following atomic percentages: 5% Cu, 35% Sb, 60 % Se (Sb_1.75_Cu_0.25_Se_3_) and 10% Cu, 30% Sb 60% (Sb_1.5_Cu_0.5_Se_3_).

Cu-doped Sb_2_Se_3_ thin films were deposited by LT-PED, with deposition parameters in the range previously defined for undoped Sb_2_Se_3_ and other selenides like CuSbSe_2_ or Cu(In,Ga)Se_2_ [14,34,40,43,44,45]. Depositions were made using a high vacuum chamber equipped with a PEBS-20 commercial source (supplied by Neocera Inc., Beltsville, MD, USA), pumped down to a base pressure of ~2.0 × 10^−4^ Pa. The pulsed e-beam was ignited at a discharge voltage between 12 kV and 18 kV. Pulse repetition-rate was fixed at 9 Hz. During the deposition process, Ar gas (5 N purity) was introduced at a pressure of about 3.0 × 10^−1^ Pa for igniting the electron beam and stabilizing the beam propagation towards the target. The films were deposited directly onto 2.5 × 2.5 cm^2^-wide commercial glass coated with Fluorine Tin Oxide (FTO) or Molybdenum (Mo). Before entering the vacuum system, the substrates were cleaned by sequentially rinsing in acetone, ethanol, and isopropyl alcohol.

The structural properties of the films, including crystalline quality and preferential orientations, were characterized by powder X-ray diffraction (XRD) measurements, performed with a Siemens D500 (Siemens, Munich, Germany) diffractometer and a Rigaku SmartLab XE diffractometer (Rigaku, Tokyo, Japan) in Bragg–Brentano geometry with Cu-Kα radiation.

The morphological and compositional analysis of the samples was made by a ZEISS Auriga Compact field-emission scanning electron microscope (FESEM-FIB, Zeiss, Oberkochen, Germany) equipped with an Oford Xplore30 energy dispersive X-ray spectrometer (EDS microanalysis, Oxford, Abingdon, UK) with Si drift detector. The electron beam acceleration was 10 kV or 20 kV for SEM imaging and 20 kV for EDS analysis.

Raman measurements were carried out using a Horiba LabRam HR Evolution micro-Raman spectrometer (Horiba, Kyoto, Japan) equipped with a confocal Olympus microscope (Olympus, Tokyo, Japan) and 10x, 50x, ULWD50x, 100x objectives (spatial resolutions of approximately 1 µm). The micro-Raman apparatus is completed by a He-Ne laser emitting at 632.8 nm, BraggRate Notch Filters, Silicon CCD + InGaAs diode array detectors, gratings 300–600–1800 lines/mm, and density filters. The spectrometer was calibrated using the standard silicon Raman peak at 520.6 cm^−1^ before each measurement. The spectra here reported were recorded using the 100x objective, for 30 s and 4 repetitions, using a 3.2% density filter. Peak fitting was carried out using a Lorentzian function.

Capacitance–voltage (C–V) and thermal admittance spectroscopy (TAS) measurements were performed using a 4192A HP (HP, Palo Alto, CA, USA) impedance analyzer at frequencies ranging from 1 kHz to 1 MHz. The amplitude of the test signal was 35 mV. The TAS spectra were recorded in the 120 ÷ 300 K temperature range while heating up. A parallel equivalent circuit model was used to obtain the measurement of both the conductance and the capacitance.

Current–voltage (I–V) characteristics of the solar cells were measured by a Keithley 2614B multimeter (Keithley, Cleveland, OH, USA) under standard test conditions using an ABET SUN 2000 solar simulator (Abet Technologies, Milford, CT, USA).

## Figures and Tables

**Table 1:** Electrical parameters of Cu-doped Sb_2_Se_3_ solar cells on Mo and FTO substrates calculated from IV characteristics under dark conditions at 300K.

Substrate Type	Ideality Factor	R_s_ (Ω cm^2^)	R_sh_ (Ω cm^2^)
Mo	1.87	0.48	2.5 × 10^5^
FTO	1.81	4.64	2.5 × 10^7^

## References

[1] Giraldo S., Jehl Z., Placidi M., Izquierdo-Roca V., Perez-Rodriguez A., Saucedo E. (2019). Progress and Perspectives of Thin Film Kesterite Photovoltaic Technology: A Critical Review. Adv. Mater..

[2] Jena A.K., Kulkarni A., Miyasaka T. (2019). Halide Perovskite Photovoltaics: Background, Status, and Future Prospects. Chem. Rev..

[3] Bouich A., Guaita J.M., Soucase B.M., Palacios P. (2022). Manufacture of High-Efficiency and Stable Lead-Free Solar Cells through Antisolvent Quenching Engineering. Nanomaterials.

[4] Punathil P., Artegiani E., Zanetti S., Lozzi L., Kumar V., Romeo A. (2022). A simple method for Ge incorporation to enhance performance of low temperature and non-vacuum based CZTSSe solar cells. Sol. Energy.

[5] Chen C., Li K.H., Tang J. (2022). Ten Years of Sb_2_Se_3_ Thin Film Solar Cells. Solar RRL.

[6] Mavlonov A., Razykov T., Raziq F., Gan J.T., Chantana J., Nishimura T., Wei H.M., Zakutayev A., Minemoto T., Zu X.T. (2020). A review of Sb_2_Se_3_ photovoltaic absorber materials and thin-film solar cells. Sol. Energy.

[7] Singh Y., Maurya K.K., Singh V.N. (2021). A review on properties, applications, and deposition techniques of antimony selenide. Sol. Energ. Mat Sol. C..

[8] Li D.B., Yin X., Grice C.R., Guan L., Song Z., Wang C., Chen C., Li K., Cimaroli A.J., Awni R.A. (2018). Stable and efficient CdS/Sb_2_Se_3_ solar cells prepared by scalable close space sublimation. Nano Energy.

[9] Zhao Y., Wang S., Li C., Che B., Chen X., Chen H., Tang R., Wang X., Chen G., Wang T. (2022). Regulating deposition kinetics via a novel additive-assisted chemical bath deposition technology enables fabrication of 10.57%-efficiency Sb_2_Se_3_ solar cells. Energy Environ. Sci..

[10] Wen X., Chen C., Lu S., Li K., Kondrotas R., Zhao Y., Chen W., Gao L., Wang C., Zhang J. (2018). Vapor transport deposition of antimony selenide thin film solar cells with 7.6% efficiency. Nat. Commun..

[11] Kumar V., Artegiani E., Kumar A., Mariotto G., Piccinelli F., Romeo A. (2019). Effects of post-deposition annealing and copper inclusion in superstrate Sb_2_Se_3_ based solar cells by thermal evaporation. Sol. Energy.

[12] Yang K., Li B., Zeng G. (2020). Sb_2_Se_3_ thin film solar cells prepared by pulsed laser deposition. J. Alloys Compd..

[13] Spaggiari G., Pattini F., Bersani D., Calestani D., De Iacovo A., Gilioli E., Mezzadri F., Sala A., Trevisi G., Rampino S. (2021). Growth and structural characterization of Sb_2_Se_3_ solar cells with vertical Sb_4_Se_6_ ribbon alignment by RF magnetron sputtering. J. Phys. D Appl. Phys..

[14] Pattini F., Rampino S., Mezzadri F., Calestani D., Spaggiari G., Sidoli M., Delmonte D., Sala A., Gilioli E., Mazzer M. (2020). Role of the substrates in the ribbon orientation of Sb_2_Se_3_ films grown by Low-Temperature Pulsed Electron Deposition. Sol. Energ. Mat Sol. C..

[15] Zhou Y., Wang L., Chen S., Qin S., Liu X., Chen J., Xue D.J., Luo M., Cao Y., Cheng Y. (2015). Thin-film Sb_2_Se_3_ photovoltaics with oriented one-dimensional ribbons and benign grain boundaries. Nat. Photonics.

[16] Wang L., Li D.L., Li K., Chen C., Deng H.X., Gao L., Zhao Y., Jiang F., Li L., Huang F. (2017). Stable 6%-efficient Sb_2_Se_3_ solar cells with a ZnO buffer layer. Nat. Energy.

[17] Wang Y.Z., Ji S., Shin B. (2022). Interface engineering of antimony selenide solar cells: A review on the optimization of energy band alignments. J. Phys. Energy.

[18] Liang G.X., Chen M.D., Ishaq M., Li X.R., Tang R., Zheng Z.H., Su Z.H., Fan P., Zhang X.H., Chen S. (2022). Crystal Growth Promotion and Defects Healing Enable Minimum Open-Circuit Voltage Deficit in Antimony Selenide Solar Cells. Adv. Sci..

[19] Fan P., Chen G.J., Chen S., Zheng Z.H., Azam M., Ahmad N., Su Z.H., Liang G.X., Zhang X.H., Chen Z.G. (2021). Quasi-Vertically Oriented Sb_2_Se_3_ Thin-Film Solar Cells with Open-Circuit Voltage Exceeding 500 mV Prepared via Close-Space Sublimation and Selenization. ACS Appl. Mater. Inter..

[20] Huang M.L., Lu S.C., Li K.H., Lu Y., Chen C., Tang J., Chen S.Y. (2021). p-Type Antimony Selenide via Lead Doping. Solar RRL.

[21] Li W.H., Li M., Hu Y.J., Cheng C.H., Kan Z.M., Yu D.Q., Leng J., Jin S.Y., Cong S.L. (2021). Enhanced performance of antimony selenide thin film solar cell using PbI_2_ as a dopant. Appl. Phys. Lett..

[22] Liang G.X., Chen X.Y., Ren D.L., Jiang X.X., Tang R., Zheng Z.H., Su Z.H., Fan P., Zhang X.H., Zhang Y. (2021). Ion doping simultaneously increased the carrier density and modified the conduction type of Sb_2_Se_3_ thin films towards quasi-homojunction solar cell. J. Mater..

[23] Stoliaroff A., Lecomte A., Rubel O., Jobic S., Zhang X.H., Latouche C., Rocquefelte X. (2020). Deciphering the Role of Key Defects in Sb_2_Se_3_, a Promising Candidate for Chalcogenide-Based Solar Cells. ACS Appl. Energy Mater..

[24] Li Y., Zhou Y., Zhu Y.N., Chen C., Luo J.J., Ma J.Y., Yang B., Wang X.J., Xia Z., Tang J. (2016). Characterization of Mg and Fe doped Sb_2_Se_3_ thin films for photovoltaic application. Appl. Phys. Lett..

[25] Rehman S., Butt F.K., Li C.B., Ul Haq B., Tariq Z., Aleem F. (2018). First-principles calculations of nitrogen-doped antimony triselenide: A prospective material for solar cells and infrared optoelectronic devices. Front. Phys..

[26] Luo X., Ren D.L., Zhang R., Wang Y.P., Chen S., Cathelinaud M., Xu Y., Qiao X.S., Zhang X.H., Fan X.P. (2022). Homogroup Bi/Sb Lattice Substitution to Enhance the Photoelectric Properties of Sb_2_Se_3_ Crystals. J. Phys. Chem C.

[27] Li J., Wang B., Liu F.Y., Yang J., Li J.Y., Liu J., Jia M., Lai Y.Q., Liu Y.X. (2011). Preparation and characterization of Bi-doped antimony selenide thin films by electrodeposition. Electrochim. Acta.

[28] Ma Y.Y., Tang B.B., Lian W.T., Wu C.Y., Wang X.M., Ju H.X., Zhu C.F., Fan F.J., Chen T. (2020). Efficient defect passivation of Sb_2_Se_3_ film by tellurium doping for high performance solar cells. J. Mater. Chem. A.

[29] Costa M.B., Lucas F.W.D., Mascaro L.H. (2018). Electrodeposition of Fe-doped Sb_2_Se_3_ thin films for photoelectrochemical applications and study of the doping effects on their properties. J. Solid State Electrochem..

[30] Ren D.L., Chen S., Cathelinaud M., Liang G.X., Ma H.L., Zhang X.H. (2020). Fundamental Physical Characterization of Sb_2_Se_3_-Based Quasi-Homojunction Thin Film Solar Cells. ACS Appl. Mater. Inter..

[31] Chen C., Li K.H., Chen S.Y., Wang L., Lu S.C., Liu Y.H., Li D.B., Song H.S., Tang J. (2018). Efficiency Improvement of Sb_2_Se_3_ Solar Cells via Grain Boundary Inversion. ACS Energy Lett..

[32] Xue D.J., Yang B., Yuan Z.K., Wang G., Liu Z., Zhou Y., Hu L., Pan D., Chen S., Tang J. (2015). CuSbSe_2_ as a Potential Photovoltaic Absorber Material: Studies from Theory to Experiment. Adv. Energy Mater..

[33] Welch A.W., Baranowski L.L., Zawadzki P., Lany S., Wolden C.A., Zakutayev A. (2015). CuSbSe_2_ photovoltaic devices with 3% efficiency. Appl. Phys. Express.

[34] Rampino S., Pattini F., Bronzoni M., Mazzer M., Sidoli M., Spaggiari G., Gilioli E. (2018). CuSbSe_2_ thin film solar cells with ~4% conversion efficiency grown by low-temperature pulsed electron deposition. Sol. Energy Mater. Sol. Cells.

[35] Fleck N., Hobson T.D.C., Savory C.N., Buckeridge J., Veal T.D., Correia M.R., Scanlon D.O., Durose K., Jäckel F. (2020). Identifying Raman modes of Sb_2_Se_3_ and their symmetries using angle-resolved polarised Raman spectra. J. Mater. Chem. A.

[36] Yan H., Xiao R., Pei Y., Yang K., Li B. (2020). Structural, electrical and optical characteristics of CuSbSe_2_ films prepared by pulsed laser deposition and magnetron sputtering processes. J. Mater. Sci. Mater. Electron..

[37] Pattini F., Bronzoni M., Mezzadri F., Bissoli F., Gilioli E., Rampino S. (2013). Dynamics of evaporation from CuGaSe2 targets in pulsed electron deposition technique. J. Phys. D Appl. Phys..

[38] Rampino S., Pattini F., Malagù C., Pozzetti L., Stefancich M., Bronzoni M. (2014). Application of a substrate bias to control the droplet density on Cu (In, Ga) Se2 thin films grown by Pulsed Electron Deposition. Thin Solid Films.

[39] Cwil M., Igalson M., Zabierowski P., Siebentritt S. (2008). Charge and doping distributions by capacitance profiling in Cu(In,Ga)Se_2_Cu(In,Ga)Se_2_ solar cells. J. App. Phys..

[40] Tang R., Chen S., Zheng Z.H., Su Z.H., Luo J.T., Fan P., Zhang X.H., Tang J., Liang G.X. (2022). Heterojunction Annealing Enabling Record Open-Circuit Voltage in Antimony Triselenide Solar Cells. Adv. Mater..

[41] Duan Z., Liang X., Feng Y., Ma H., Liang B., Wang Y., Luo S., Wang S., Schropp R.E.I., Mai Y. (2022). Sb_2_Se_3_ Thin-Film Solar Cells Exceeding 10% Power Conversion Efficiency Enabled by Injection Vapor Deposition Technology. Adv. Mater..

[42] Hegedus S.S., Shafarman W.N. (2004). Thin-film solar cells: Device measurements and analysis. Prog. Photovolt. Res. Appl..

[43] Mazzer M., Rampino S., Spaggiari G., Annoni F., Bersani D., Bissoli F., Bronzoni M., Calicchio M., Gombia E., Kingma A. (2017). Bifacial CIGS solar cells grown by low temperature pulsed electron deposition. Sol. Energy Mater. Sol. Cells.

[44] Cavallari N., Pattini F., Rampino S., Annoni F., Barozzi M., Bronzoni M., Gilioli E., Gombia E., Maragliano C., Mazzer M. (2017). Low temperature deposition of bifacial CIGS solar cells on Al-doped Zinc Oxide back contacts. Appl. Surf. Sci..

[45] Mazzer M., Rampino S., Gombia E., Bronzoni M., Bissoli F., Pattini F., Calicchio M., Kingma A., Annoni F., Calestani D. (2016). Progress on Low-Temperature Pulsed Electron Deposition of CuInGaSe_2_ Solar Cells. Energies.

